# Oral health care knowledge among Phenylketonuria patients in the Latvian population

**DOI:** 10.1016/j.ymgmr.2024.101167

**Published:** 2024-11-27

**Authors:** Iveta Abola, Nikola Anna Intlere, Anda Brinkmane, Sabine Laktina, Agnese Zarina, Lauma Vasilevska, Ingus Skadins, Georgijs Moisejevs, Linda Gailite, Madara Auzenbaha

**Affiliations:** a Department of Conservative Dentistry and Oral Health, Rīga Stradiņš University, LV-1007 Rīga, Latvia; b Scientific Laboratory of Molecular Genetics, Rīga Stradiņš University, LV-1007 Rīga, Latvia; c Clinic of Medical Genetics and Prenatal Diagnostics, Children's Clinical University Hospital, LV-1004 Rīga, Latvia; d Department of Biology and Microbiology, Rīga Stradiņš University, LV-1007 Rīga, Latvia; e Jekabpils Regional Hospital, LV-5201, Jekabpils, Latvia; f European Reference Network for Rare Hereditary Metabolic Disorders, Latvia

**Keywords:** Dental health education, Dental status, Oral health, Phenylketonuria

## Abstract

**Background:**

Phenylketonuria (PKU) is an autosomal recessive inherited disorder of phenylalanine (Phe) metabolism that results from a deficiency of phenylalanine hydroxylase (PAH). Patients with PKU rely on amino acid mixtures and low-protein diets, which often exhibit an acidic nature and pose various challenges to oral health. The objective of the study was to evaluate oral care habits of PKU patients in Latvia and the impact of the recommendations developed on improving oral care.

**Materials and methods:**

In this study, during a one-month interval before and after the implementation of oral hygiene recommendations, questionnaires were distributed to all patients with PKU diagnosed in Latvia, with a response rate of 78 % (79 of 101).

**Results:**

The group older and 18 years of age showed a poorer understanding of oral care even after receiving recommendations, 82 % brushing their teeth twice a day (92 % in the group <18 years of age), continuing 57 % rinsing their mouth after using amino acid formula (75 % in the younger group). Significant improvements were observed only in the respondent group younger than 18 years of age - including increases in toothbrushing twice a day by 25 % (*p* = 0.001), dental flossing by 23 % (*p* = 0.001), mouth rinsing after amino acid-based formula by 13 % (*p* = 0.020).

**Conclusion:**

This study concludes that PKU patients older and 18 years of age have a poor understanding of maintaining oral hygiene and the use of the necessary supplements to improve it. Activities are needed in the future that would regularly remind and motivate PKU patients to take care of their oral health.

## 1 Introduction

Phenylketonuria (PKU) is a hereditary disorder of metabolism, with an autosomal recessive pattern of inheritance [1]. PKU treatment consists mainly of lowering proteins in the diet to ensure that phenylalanine (Phe) does not accumulate and have a toxic impact [2]. Patients diagnosed with classic PKU have strict dietary requirements, allowing a restricted intake of approximately 5–6 g of natural proteins per day. Furthermore ingestion of artificial sweeteners, aspartame, and its derivatives is not allowed due to their Phe composition [3]. To ensure appropriate growth and caloric intake, people with PKU rely on specialized amino acid mixtures as substitutes for natural proteins [4,5]. PKU patients should be recommended to rinse their mouth with water immediately after consuming the PKU formula to counteract the acidity in their oral cavity [6]. The increased acidity in the oral cavity too often or for prolonged periods results in demineralization of the tooth structures. This process is known as dental caries, and all PKU patients are susceptible to higher levels of caries activity due to their dietary needs [7]. During periods of illness, it is recommended that PKU patients consume additional high-energy snacks, for example those containing glucose polymers, which can result in recurrent drops in salivary pH levels, which, combined with insufficient oral hygiene, result in an increased incidence of caries [8].

Oral health is an essential part of general health and maintaining good oral hygiene is vital to sound general health [9]. The two most common oral diseases are dental caries and periodontal diseases, and these are the main causes of tooth loss. Biofilm is necessary for the development of these oral diseases [10]. The elimination of plaque is essential for the maintenance of good oral hygiene and oral health. Brushing your teeth twice a day with fluoridated toothpaste and flossing between teeth can help reduce plaque accumulation [11]. Daily oral practice should also include antimicrobial mouthwash to reduce the amount of plaque [12,13]. In addition, regular dental visits and professional dental hygiene procedures help to maintain oral health [14].

Periodontitis, a chronic multifactorial inflammation of periodontal tissue, is mainly caused by the accumulation of plaque and calculus on the teeth, leading to gum irritation and subsequent inflammation [15]. This inflammatory process results in the development of gingivitis, an early reversible form of periodontitis [16]. By consistently practicing good oral hygiene, patients can effectively manage and reverse gingivitis, preventing progression to more severe forms of periodontitis [17].

In most cases, incorrect and/or irregular teeth cleaning and lack of proper oral hygiene are the direct cause of periodontal illness manifestations [18,19].

Consequently, based on objective findings from the recent study on the dental status and periodontal health PKU patients in Latvia, overall, aged 12 and older had a higher prevalence of carious teeth than the control group, and the values of oral hygiene indices were significantly higher [6,7,20]. Most of the patients with PKU struggled with maintaining good oral hygiene, which could lead to plaque accumulation and, over time, to periodontal disease [16]. To meet energy requirements, PKU patient's diets are often high in carbohydrates, which are taken throughout the day. This kind of diet is highly cariogenic and can be associated with a higher risk of caries development [21].

Hence, it was deemed necessary to develop customized oral health recommendations for PKU patients in Latvia in the national language, to improve oral health outcomes and reduce the risk of dental caries and periodontal disease.

The study's null hypothesis is that the development of targeted recommendations does not improve the knowledge of PKU patients about the correct daily care of their oral cavity.

The study aimed to analyse oral hygiene habits through a questionnaire that examines the actions that patients with PKU take and the tools they use in their daily oral hygiene routines before and after receiving tailored recommendations, to evaluate whether patients with PKU practically implemented these recommendations in their daily hygiene routine.

## 2 Material and methods

### 2.1 Ethics approval

The present study obtained research approval from the Central Medical Ethics Committee prior to data collection with permission (no. 1/10–03-26). The study was conducted in the Clinic of Medical Genetics and Prenatal Diagnostics at the Children's Clinical University Hospital according to the Declaration of Helsinki. This study has been designed to meet STROBE guidelines [22].

### 2.2 Inclusion and exclusion criteria

The number of patients with PKU diagnosed in Latvia is 122. 101 of the patients are reachable and at least occasionally tested for Phe levels, of which 46 patients are older and 18 years of age. An invitation to participate in the study was distributed to all 101 patients, of which 79 applied (78 %). The inclusion criteria of PKU patients ≥ and < 18 years of age were biochemically and molecularly confirmed PKU diagnosis. The exclusion criteria for patients with PKU were the following: unknown place of residence and contact information, lost in follow-up questioning, or unwillingness to participate in the study.

### 2.3 Survey

48 PKU patients <18 years of age and 31 PKU patients ≥18 years of age were included in this cross-sectional study from 2022 to 2023. The structured survey written in Latvian was completed and sent to experts in a field for necessary feedback on the content, sensitivity, and standard settings. The survey included ten questions about oral hygiene procedures, the frequency of tooth brushing, flossing, tongue cleaning, the frequency of visits to the dentist and dental hygienist, the use of aspartame-free toothpaste, the rinsing of the mouth each time after the intake of specialized amino acid mixtures, and one question about the level of education of the individual (Questions on Supplementary S1). The questionnaire began with a brief explanation of the study's purposes and asked the respondents to answer as sincerely as possible. The questionnaire was validated on 15 non-PKU individuals, from health care professionals and the general population, and modifications were made necessary to resolve ambiguities. People who participated in the pilot study were not included in the main study. All questions could give only one answer from the multiple-choice questions. The study was carried out by trained medical personnel who debriefed the questionnaire over the phone in the style of an interview. For patients <18 years of age, their parents or legal guardians filled out questionnaires. Patients with PKU received written recommendations consisting of 13 tasks that aim to improve oral health understanding and prevent the increase in Phe levels, by using toothpaste and chewing gum without aspartame (Supplementary S2). The recommendations were created by dentists and rare disease specialists. After a month, each participant was contacted by phone and a repeated survey was conducted. In the group ≥18 years of age three patients could not be contacted again.

### 2.4 Statistical analysis

Statistical analysis was performed using SPSS for Windows (SPSS, version R 4.1.2, SPSS Inc., Chicago, IL, USA) and Jamovi v.2.3.(www.jamovi.org). For clinical variables and survey responses, descriptive statistics, mean and standard deviation calculation were used. The mean values were compared using the Kruskal -Walli's test. Changes in responses before and after the survey were calculated using the Wilcoxon signed rank test. A *p*-value of <0.05 was considered as an indicator of a statistically significant result. Statistical power for the analysis was not calculated as nonrandomised patient selection was used, as all PKU patients were asked to participate in the study.

## 3 Results

In the group less than 18 years old, 48 patients (mean age 6.2 ± 5.88 years), 27 male and 21 female participants were enrolled for survey. The number PKU patients older and 18 years of age who participated in the survey was 31 (mean age 30.37 ± 7.96 years), 12 male and 19 female. Survey results for both age groups were compared before and after receiving recommendations shown in Table 1.

**Table 1:** Daily oral hygiene habits in study groups before and after receiving tailored recommendations.

		PKU patients Younger than 18 years of age						PKU patients Older and 18 years of age					
		Before Recommendations		After Recommendations			⁎*p*-Value	Before Recommendations		After Recommendations			⁎*p*-Value
		*n* = 48	%	n = 48	%	Improvement⁎⁎ %		*n* = 31	%	*n* = 28	%	Improvement %	
How often do you/your child brush your / your child's teeth?	Twice a day	32	66	44	92	25	0.001	23	74	23	82	8	1.000
	Once a day	13	27	4	8			8	26	5	18		
	Less than daily	3	6	0	0			0	0	0	0		
Do you/your child use dental floss daily?	Yes	8	17	19	40	23	0.001	12	39	9	32	−7	1.000
	No	40	83	29	60			19	61	19	68		
How often do you/your child visit a dentist?	Twice a year	7	15	20	42	27	<0.001	5	16	8	29	13	0.149
	Once a year	21	44	27	56			14	45	10	36		
	Rarely	20	41	1	2			12	39	10	36		
How often do you/your child go to a dental hygienist?	Twice a year	8	17	16	33	17	<0.001	4	13	8	29	16	0.072
	Once a year	17	35	26	54			10	32	6	21		
	Rarely	23	48	6	12			17	55	14	50		
Do you/your child consistently rinse your mouth with water after each use of an amino acid-based formula?	No	18	37	12	25			15	48	12	43		
	Together replies Yes and descriptive	30	63	36	75	13	0.020⁎⁎⁎	16	52	16	57	6	1.000

⁎ Wilcoxon signed rank test.

⁎⁎ showed improvement in the desirable outcome.

⁎⁎⁎ compared to those who do not rinse the mouth after amino acid-based formula.

It is important to mention that 90 % of the respondents in both age groups agree that the recommendations provided would improve their/their children's daily oral care habits, although a statistically significant improvement was observed only in younger patients (Table 1).

**Fig. 1:**
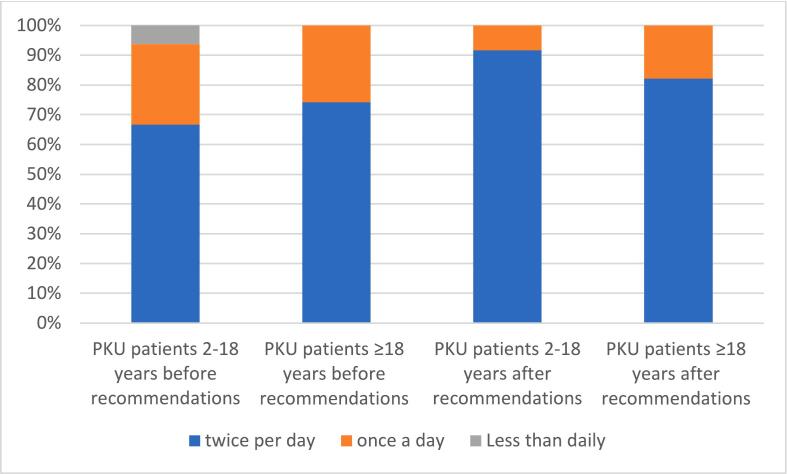
Tooth brushing frequency in PKU patients in two age groups before and after receiving the recommendations.

The figure illustrates the comparison between age groups in tooth brushing frequency before and after the recommendations (Fig. 1).

According to questionnaires, 87 % PKU patients <18 years of age and 42 % ≥ 18 years of age use toothpaste without aspartame, but one month after the recommendations and reminders of toothpaste brands containing aspartame, the survey results were different in the group ≥18 years of age, with 60 % using toothpaste without aspartame, with an improvement of 18 %.

To test their knowledge, participants were asked to name the toothpaste they used. Several brands were reported, which did not contain aspartame, one mentioned fluoride toothpaste, some participants could not recall the name of the toothpaste used.

In patients older than 14 years, a question was included to determine whether tongue cleaning was a part of their daily oral hygiene practice, 24 patients (63.2 %) reported also cleaning the tongue. The survey after recommendations included a question on the factors that contribute to non-compliance with oral health guidelines and recommendations, 2 (7 %) of the older group mentioned financial problems, 17 (35 %) in the younger group, and 10 (36 %) in the older group admitted to trying but with no results, but 11 (23 %) (in younger group) and 5 (18 %) in the older group reported that no guidelines or recommendations are needed.

The level of education in this group was the following, 20 % of adult patients with PKU have a higher education and 23 % needed special education, the rest received general secondary or vocational secondary education.

## 4 Discussion

This study shows previously underestimated problems with PKU patients' insufficient dental hygiene habits, although it is known that this patient group due to dietary restrictions has more risk factors for oral health leading to more carious teeth [6]. One of the possible reasons could be the lack of targeted and probably written recommendations; in this study we surveyed PKU patients or for minors their parents about oral habits, then provided targeted recommendations and repeated the survey.

Dental caries (tooth decay) affects most people worldwide, causing pain, difficulties eating and speaking, low self-esteem, tooth loss, and the need for surgery. PKU patients are at higher risk of having carious teeth compared to the general population; this is observed not only in Latvia [6] but also in other countries, such as Germany and Turkey [20,23]. As dental plaque is the primary cause of dental caries and periodontal disease, daily oral hygiene procedures and plaque removal are important for oral health [24].

Tooth brushing is considered a fundamental self-care behaviour for the maintenance of oral health, brushing twice a day has become a social norm [25]. The fact that only 74 % of PKU patients ≥18 years of age and 66 % < 18 years of age brush their teeth twice a day is a proof that knowledge and motivation about oral hygiene remain low for PKU patients in Latvia. Insufficient oral hygiene habits are prevalent not only in PKU patients, but also in the general population, and that is why the World Health Organization is addressing the importance of oral hygiene [https://www.who.int/health-topics/oral-health] [26]. The last report from Latvia in 2023 showed that 45 % of 12-year-old and 51 % of 15-year-old children brush their teeth two or more times a day. Although 5 % of 12-year-olds and 3 % of 15-year-olds do not brush their teeth at all [27]. In the UK, the baseline data from the Brushing RemInder 4 Good oral HealTh (BRIGHT) trial reported that 69,8 % of secondary school pupils brush their teeth twice a day [28]. In France, the proportion of adolescents brushing their teeth twice a day in 2024 reached 78.8 % [29]. In a study from Finland, survey data for people aged 15 to 64 years (*n* = 119,665) reported that brushing twice a day contributes to 66 % and once per day 95 % [30]. Data analysis from other populations shows that Latvia generally has poorer adherence to healthy oral habits, but in PKU patients it has a greater effect due to diet restrictions. In this context, it is important to emphasise that oral care recommendations should also be provided to the general population in order to improve oral health status and to improve home oral care.

Using floss or interdental brushes in addition to brushing is also very important to remove plaque between two adjacent teeth. It may reduce gingivitis more than toothbrushing alone [31]. It is strongly recommended to use daily interdental cleaning devices and mouthwash, in addition to brushing, to prevent and control periodontal diseases and dental caries [32]. According to the questionnaire, only 39 % of PKU patients ≥18 years of age and 17 % of PKU patients <18 years of age use dental floss daily. It makes one wonder about the knowledge, role, and modelling of daily hygiene procedures by parents, as well as the constant reminders by the dentist of proper daily oral health care. A daily mouthwash is recommended for PKU patients to reduce dental plaque. The results concluded that still many patients can be introduced to natural mouthwashes, as more than 80 % of PKU patients have not heard about them or did not include them in their daily hygiene procedures. It is not possible to compare data from other populations with PKU patients, as they have published information about the frequency of brushing teeth, but missing information on mouth washing after use of amino acid formula and flossing [7,20,23].

Neves et al. in their study demonstrated the crucial role of the family in the oral health of their children [33]. Parents of children with PKU prioritize maintaining optimal general health and daily functioning rather than focusing on prevention of oral disease. Children with chronic medical conditions and their families face many pressures and are often delayed in seeking dental care as it is simply not a priority [34]. By motivating parents and regularly reminding them of the oral care of children, this study confirmed that parents play an important role in maintaining their children's oral health, as significant improvement in oral hygiene after receiving recommendations was observed in patients under 18 years of age, suggesting the influence of parents on the oral care of their children.

Another important variable that determines the overall well-being of PKU patients, including their oral health, is the level of education or the education of the parents. It has been reported that the higher the level of education of parents, the greater the understanding of the disease, and the greater the support for the affected child [35,36]. Regarding adults with PKU, it has been proposed that the increased risk of oral health problems may be due to social burdens [20,37]. It is important to mention that when interviewing PKU patients, the level of education was determined only for adult patients, but not for parents and the results showed that 20 % of adult PKU patients have higher education and 23 % have special education. Patients with higher education are more likely to practice oral hygiene, brush their teeth more often, and having regular dental checkups [38]. This study showed that in the ≥18-year-old group, there were no significant improvements in daily oral care habits even after receiving recommendations.

Through consultations and patient interviews, a notable observation emerged, indicating that PKU patients not only exhibit poor oral health compared to the control group. Patients reported limited awareness among dentists and dental hygienists of the specific challenges associated with PKU and its impact on oral health and hygiene.

Patients with PKU must carefully select their toothpaste considering their formulation. Toothpaste must not contain aspartame, a sweetener [39,40]. The answers of the questionnaire show that 87 % of PKU patients <18 years of age and 42 % ≥ 18 years of age claim that the toothpaste they use does not contain aspartame. Unfortunately, this leads to the conclusion that PKU patients and their parents do not pay attention to such important details and recommendations about proper daily oral care, using toothpastes without aspartame.

The recommendations included information about saliva stimulation. Saliva is an important factor in a wide range of oral functions, such as mastication, swallowing, antimicrobial activity, and cleaning action [41]. An investigation of the oral health of PKU patients in Latvia revealed that 60 % of PKU patients had a lower basal saliva secretion rate compared with healthy counterparts [6]. Saliva influences oral health both through its nonspecific physiochemical properties and through more specific effects [41]. Salivation can be induced by gustatory or masticatory stimulation, such as using chewing gum [42]. Other desirable properties of chewing gums are adequate buffer capacity and the use of non-cariogenic sweeteners such as xylitol, sorbitol, and mannitol. These sweeteners will not result in an increase in the incidence of caries due to fermentation by oral microorganisms but will instead decrease the caries activity [43,44]. PKU and their parents should know that chewing gum must not contain aspartame [45].

Dental and dental hygienist visits for caries prevention and gingival health are low. In the literature, the most recommended revisiting period for dental care is 6 months [46]. Dental professionals are convinced that frequent examinations allow disease to be detected and treated in time and preventive interventions to be delivered [47]. A clinical guideline recommends that the longest period between examinations for both children and adults should be 12 months; For adults who maintain good oral health and appropriate home care habits, this can be extended to 24 months [47,48].

It is important that the metabolic team, dentists, general practitioners, and psychologists encourage, motivate, and support PKU patients to follow the recommendations of oral hygiene procedures, as 90 % of PKU patients < and ≥ 18 years of age believe that this will improve their oral health and sequentially overall quality of life. To increase the awareness among healthcare professionals about oral hygiene habits in PKU patients, oral care recommendations for PKU patients were published in a local journal [49].

The limitation of the study should certainly be mentioned in the small number of nonrandomised patients, but it should be noted that it includes in total of 79 patients, which is 78 % of all PKU patients in Latvia who regularly perform counter examinations. It would be valuable to repeat the survey after 3 years to see the long-term effect of the recommendations provided. It should be noted that there is low self-motivation, weak response of PKU patients, reluctance to go to dental examinations and answer questionnaires. Patients with PKU should be addressed at all times and motivated to take care of their health.

Rare conditions are not typical areas of focus, especially in terms of prevalence. Ordinarily, the pertaining to patients with PKU in the dental profession focuses on the actionable steps that a medical specialist can take within their practice or solely discusses oral health manifestations [7]. Additionally, future research is suggested to place greater emphasis on preventive measures customized to the oral health needs of patients with PKU with the aim of reducing the occurrence and severity of oral health complications. Data collection methods may differ, leading to a range of outcomes [50]. In this study, the chosen data collection method involved a survey before and after implementing preventive measures. The fact that the questionnaire was conducted by phone should also be mentioned as a limitation. Therefore, the proposed method for future studies is to conduct research in a way that allows long-term patient monitoring and increased in-person visits throughout the study period. Referring to challenges in data acquisition, an issue to consider is patient bias. Any survey conducted is susceptible to the tendency of the patient to ignore complete candour in their responses. This concern can be effectively mitigated by incorporating personal consultations following the survey, thus reducing the likelihood of patient bias, or additionally free toothpastes, dental floss, and mouthwashes could be distributed.

This study highlighted the attitude of PKU patients and the lack of knowledge of oral hygiene, especially in the group older and 18 years of age. This problem was previously underestimated. Future activities should be carried out, as only recommendations alone do not reach the goal.

## 5 Conclusions

The study shows that patients with PKU, especially those ≥18 years old, have a poor understanding of the importance of good oral hygiene habits, the use of toothpaste without aspartame, and regular mouth washing after using the amino acid formula. This is particularly important in the case of PKU for two reasons. One is specific diet requirements - amino acid mixtures and usage of acidic fruits e.g. oranges, apples that might lead to higher tooth damage, this is the same in the case of the general population, and possible aspartame content in chewing gums, toothpaste, and mouthwashes. Regular prophylactic observation and educational activities, e.g. repetition of recommendations, by experienced dentists and hygiene specialists, are strongly recommended to raise awareness for oral health. This study created and distributed customized recommendations for patients with PKU and their families to take preventive actions every day to help avoid complications with respect to oral health in the local language. An important step in the health care of rare diseases would be to prioritize state paid dental treatments for PKU patients and professional dental hygiene as only recommendations alone do not reach the goal.

## Funding

No external funding.

## Institutional review board statement

The present study obtained research approval from the Central Medical Ethics Committee prior to data collection under permission (nr. 1/10–03-26). The study was conducted according to the Declaration of Helsinki.

## Informed consent statement

All participants and their parents provided their informed written consent.

## CRediT authorship contribution statement

**Iveta Abola:** Writing – original draft, Investigation, Data curation, Conceptualization. **Nikola Anna Intlere:** Data curation. **Anda Brinkmane:** Investigation. **Sabine Laktina:** Data curation. **Agnese Zarina:** Data curation. **Lauma Vasilevska:** Data curation. **Ingus Skadins:** Validation. **Georgijs Moisejevs:** Investigation. **Linda Gailite:** Project administration. **Madara Auzenbaha:** Writing – review & editing, Methodology.

## Declaration of competing interest

The authors declare that they have no conflict of interest.

## Data Availability

Data will be made available on request.

## Appendix A Supplementary data

Supplementary material Image 1

## References

[1] Lugovska R., Vevere P., Andrusaite R., Kornejeva A. (1999). Newborn screening for PKU and congenital hypothyroidism in Latvia. Southeast Asian J. Trop. Med. Public Health.

[2] Van Wegberg A.M.J. (2017). The complete European guidelines on phenylketonuria: diagnosis and treatment. Orphanet J. Rare Dis..

[3] Maler V. (2023). Aspartame and phenylketonuria: an analysis of the daily phenylalanine intake of aspartame-containing drugs marketed in France. Orphanet J. Rare Dis..

[4] Elhawary N.A. (2022). Genetic etiology and clinical challenges of phenylketonuria. Hum. Genomics.

[5] MacDonald A. (2020). PKU dietary handbook to accompany PKU guidelines. Orphanet J. Rare Dis..

[6] Abola I., Emulina D.E., Skadins I., Brinkmane A., Gailite L., Auzenbaha M. (2022). Dental status and periodontal health of patients with phenylketonuria in Latvia. Acta Stomatol. Croat..

[7] Ballikaya E. (2020). Oral health status of children with phenylketonuria. J. Pediatr. Endocrinol. Metab..

[8] Chan D.C.N. (1997). Essentials of dental caries (2nd. ed.). J. Am. Dent. Assoc..

[9] Bokhari S.A.H., Khan A.A. (2009). Growing burden of noncommunicable diseases: the contributory role of oral diseases, eastern Mediterranean region perspective. East Mediterr. Health J..

[10] Sanz M. (2017). Role of microbial biofilms in the maintenance of oral health and in the development of dental caries and periodontal diseases. Consensus report of group 1 of the joint EFP/ORCA workshop on the boundaries between caries and periodontal disease. J. Clin. Periodontol..

[11] Rimondini L., Zolfanelli B., Bernardi F., Bez C. (2001). Self-preventive oral behavior in an Italian university student population. J. Clin. Periodontol..

[12] Erbe C., Klukowska M., Timm H.C., Barker M.L., Van Der Wielen J., Wehrbein H. (2019). A randomized controlled trial of a power brush/irrigator/mouthrinse routine on plaque and gingivitis reduction in orthodontic patients. Angle Orthod..

[13] Arduino P.G. (2020). Effect of two different alcohol-free chlorhexidine formulations in mouthrinses on the immediate postoperative period for oral mucosal biopsies. J. Oral Sci..

[14] Zheng S., Zhao L., Ju N., Hua T., Zhang S., Liao S. (2021). Relationship between oral health-related knowledge, attitudes, practice, self-rated oral health and oral health-related quality of life among Chinese college students: a structural equation modeling approach. BMC Oral Health.

[15] Kwon T.H., Lamster I.B., Levin L. (2021). Current concepts in the Management of Periodontitis. Int. Dent. J..

[16] Yu N., Van Dyke T.E. (2020). Periodontitis: a host-mediated disruption of microbial homeostasis. Curr. Oral Health Rep..

[17] (2014). Color Atlas of Dental Hygiene: Periodontology.

[18] Ericsson J.S., Abrahamsson K.H., Östberg A.L., Hellström M.K., Jönsson K., Wennström J.L. (2009). Periodontal health status in Swedish adolescents: an epidemiological, cross-sectional study. Swed. Dent. J..

[19] Musić L., Par M., Peručić J., Badovinac A., Plančak D., Puhar I. (2021). Relationship between halitosis and periodontitis: a pilot study. Acta Stomatol. Croat..

[20] Bingöl A.C. (2023). Dietary and metabolic effects on the oral status of patients with phenylketonuria: a nation-based cross-sectional study. Clin. Oral Investig..

[21] Kilpatrick N.M., Awang H., Wilcken B., Christodoulou J. (1999). The implications of phenylketonuria on oral health. Pediatr. Dent..

[22] Cuschieri S. (2019). The STROBE guidelines. Saudi J Anaesth.

[23] Ghasemi N., Namvar M.A., Mansori K. (2023). Evaluation of decayed, missing due to caries, and filled teeth Index in children with phenylketonuria in comparison to Normal population. Turkish Arch. Pediatr..

[24] Ebrahimipour S., Bijari S., Talebi F., Darmiani S. (2022). The effect of oral health education on dental plaque and gingival index of female students by the virtual reality simulation method. Int. J. Med. Dent..

[25] Kumar S., Tadakamadla J., Johnson N.W. (2016). Effect of toothbrushing frequency on incidence and increment of dental caries: a systematic review and meta-analysis. J. Dent. Res..

[26] World Health Organization (2021). Seventy-fourth world health assembly: resolution on Oral health. Lancet.

[27] Maldupa I., Uribe S.E., Rivare-Palena U., Stamere U., Brinkmane A., Senakola E. (2023). Mutes veselības pētījums skolēniem Latvijā 2023 (engl. Oral health study for schoolchildren in Latvia 2023). https://ppdb.mk.gov.lv/wp-content/uploads/2023/09/Mutes_veselibas_petijums_zinojums_2023.pdf.

[28] Marshman Z. (2023). The oral health of secondary school pupils: baseline data from the brushing RemInder 4 good oral HealTh (BRIGHT) trial. Br. Dent. J..

[29] De Grado G.F. (2021). Changes in tooth brushing frequency and its associated factors from 2006 to 2014 among French adolescents: Results from three repeated cross sectional HBSC studies. PLoS One.

[30] Raittio E., Helakorpi S., Suominen A.L. (2021). Age-Period-Cohort Analysis of Toothbrushing Frequency in Finnish Adults: Results From Annual National Cross-Sectional Surveys From 1978 to 2014. Int. Dent. J..

[31] Fleming E.B., Nguyen D., Afful J., Carroll M.D., Woods P.D. (2018). Prevalence of daily flossing among adults by selected risk factors for periodontal disease—United States, 2011–2014. J. Periodontol..

[32] Worthington H.V. (2019). Home use of interdental cleaning devices, in addition to toothbrushing, for preventing and controlling periodontal diseases and dental caries. Cochrane Database Syst. Rev..

[33] Neves É.T.B. (2020). The impact of oral health literacy and family cohesion on dental caries in early adolescence. Community Dent. Oral Epidemiol..

[34] Moursi A.M., Fernandez J.B., Daronch M., Zee L., Jones C.L. (2010). Nutrition and oral health considerations in children with special health care needs: implications for oral health care providers. Pediatr. Dent..

[35] Castro I.P.S., Borges J.M., Chagas H.A., Tibúrcio J., Starling A.L.P., De Aguiar M.J.B. (2012). Relationships between phenylalanine levels, intelligence and socioeconomic status of patients with phenylketonuria. J. Pediatr..

[36] Tadakamadla S.K., Tadakamadla J., Kroon J., Lalloo R., Johnson N.W. (2020). Effect of family characteristics on periodontal diseases in children and adolescents—a systematic review. Int. J. Dent. Hyg..

[37] Cazzorla C. (2018). Living with phenylketonuria in adulthood: the PKU ATTITUDE study. Mol. Genet. Metab. Report..

[38] Minervini G., Franco R., Marrapodi M.M., Fiorillo L., Cervino G., Cicciù M. (2023). The association between parent education level, oral health, and oral-related sleep disturbance. An observational crosssectional study. Eur. J. Paediatr. Dent..

[39] Cury J.A., Tenuta L.M.A. (2014). Evidence-based recommendation on toothpaste use. Braz. Oral Res..

[40] Hu S., Lai W.P.B., Lim W., Yee R. (2021). Recommending 1000 ppm fluoride toothpaste for caries prevention in children. Proc. Singapore Healthc..

[41] Dodds M.W.J., Johnson D.A., Yeh C.K. (2005). Health benefits of saliva: a review. J. Dent..

[42] Olsson H., Spak C.J., Axéll T. (1991). The effect of a chewing gum on salivary secretion, oral mucosal friction, and the feeling of dry mouth in xerostomic patients. Acta Odontol. Scand..

[43] Kandelman D., Gagnon G. (1987). Clinical results after 12 months from a study of the incidence and progression of dental caries in relation to consumption of chewing-gum containing xylitol in school preventive programs. J. Dent. Res..

[44] Kashket S., Yaskell T., Lopez L.R. (1989). Prevention of sucrose-induced demineralization of tooth enamel by chewing sorbitol gum. J. Dent. Res..

[45] Wu Y.F. (2022). Xylitol-containing chewing gum reduces cariogenic and Periodontopathic Bacteria in dental plaque—microbiome investigation. Front. Nutr..

[46] Fee P.A., Riley P., Worthington H.V., Clarkson J.E., Boyers D., Beirne P.V. (2022). Recall intervals for oral health in primary care patients: a Cochrane review. Dent. Cadmos.

[47] National Institute for Clinical Excellence (2004).

[48] Wang N.J., Källestål C., Petersen P.E., Arnadottir I.B. (1998). Caries preventive services for children and adolescents in Denmark, Iceland, Norway and Sweden: strategies and resource allocation. Community Dent. Oral Epidemiol..

[49] Abola I., Brinkmane A., Gailite L., Auzenbaha M. (2023). Mutes un zobu veseliba fenilketonurijas pacientiem (engl. Oral and dental health in patients with phenylketonuria). Latv. Arsts.

[50] Lefever S., Dal M., Matthíasdóttir Á. (2007). Online data collection in academic research: advantages and limitations. Br. J. Educ. Technol..

