# Supplementary material for: Oral health care knowledge among Phenylketonuria patients in the Latvian population
Source: Mol Genet Metab Rep. 2024 Nov 27;41:101167. doi: 10.1016/j.ymgmr.2024.101167 (PMC11638625; doi:10.1016/j.ymgmr.2024.101167)
Supplement: Supplementary file 1 — Supplementary material [file mmc1.docx]

**Supplementary S1**

Questionnaire on daily oral hygiene for PKU patients < 18 years of age (filled out by the parents of the patient)

1. Age
2. Sex
3. Frequency of tooth brushing

Once/Twice/Less than once

1. Does your child use toothpaste without aspartame?

Yes/No

1. Does your child use a mouthwash?

Yes/No

1. Does your child practice interdental cleaning?

Yes/No

1. How often does your child visit the dentist?

Twice a year/Once a year/Less than once a year

1. How often does your child visit a dental hygienist?

Twice a year/Once a year/Less than once a year

1. Does your child rinse his mouth every time after consuming the PKU formula?

Yes/No/Other (water)

Questionnaire on daily oral hygiene for PKU patients ≥ 18 years of age

1. Age
2. Sex
3. Your level of education:

Higher education/ secondary or vocational secondary education/ special education

1. Frequency of tooth brushing

Once/Twice/Less than once

1. Do you use a toothpaste without aspartame?

Yes/No

1. Do you use a mouthwash?

Yes/No

1. Do you perform interdental cleaning?

Yes/No

1. Do you clean the tongue?

Yes/No

1. How often do you visit a dentist?

Twice a year/Once a year/Less than once a year

1. How often do you see a dental hygienist?

Twice a year/Once a year/Less than once a year

1. Do you rinse your mouth every time after consuming the PKU formula?

**Supplementary S2**

Recommendations for Oral Health

Maintenance of correct and regular oral health plays a vital role in maintaining optimal dental and gum health.

**Daily regimen:**

1) Teeth brushing twice a day: once in the morning (after breakfast) and once in the evening (before bedtime).

2) Teeth brushing should be performed with a toothpaste that does not contain aspartame. Instead, choose a toothpaste with fluoride content ranging from 1000 ppm to 1450 ppm, depending on your age group:

*For children aged 0 to 2, use a rice grain-sized amount of toothpaste with a fluoride concentration of 1000 ppm.*

*For children aged 2 to 6, a pea-sized amount of toothpaste with a fluoride concentration of 1000 ppm.*

*For individuals aged 6 and over, an amount of toothpaste equivalent to half the length of the toothbrush bristle part, with a fluoride concentration of 1450 ppm.*

3) Teeth brushing should be performed with a soft toothbrush, indicated by the 'soft' or 'ultrasoft' label on the packaging. It is recommended to opt for a compact bristle head toothbrush or an electronic variant.

4) After brushing your teeth, it is advised not to rinse with water and instead to emit excess toothpaste by spitting.

5) Children aged 10 and above are responsible for independently brushing teeth, while those under 10 years of age require parental assistance.

6) To ensure thorough cleaning of the interdental spaces, it is recommended to use dental floss or an irrigator, suitable for both deciduous and permanent teeth.

7) Tongue cleaning for adults (up to 18 years old) in the morning after brushing the teeth (with a toothbrush, a tongue scraper, or by using a spoon and pulling it from the roof of the tongue to the tip of it.

8) Salivary stimulation with chewing gums that do not contain aspartame. The mechanical action of chewing stimulates saliva production, aiding in the effective removal of dental plaque through its cleaning action.

9)After consuming an amino acid mixture, it is recommended to rinse the mouth with plain water.

10) If recommended by a dentist, mouthwash containing 0.05 or 0.09% chlorhexidine is used. Rinse with 10 mL of ready-to-use liquid or until reaching the marked line on the bottle cap, for a duration of 30 s, twice daily, 30 min after tooth brushing. After mouthwash use, it is recommended to avoid rinsing with water and instead expel excess liquid by spitting [51];

11) The implementation of mouthwash containing antimicrobial properties [52]; Conducting rinses with saltwater, coconut oil, or plant-based mouthwash (containing Aloe Vera, Echinacea, Camomile, grapefruit seed oil) 2–3 times a day, limiting the duration to 1 month and allowing a 3-month interval between usage cycles. After mouthwash application, it is recommended not to rinse with water.

12) Scheduled dental check-ups are advised every six months or twice yearly as part of a comprehensive preventive approach to oral health.

13) Routine professional oral hygiene procedures performed by a dental hygienist at intervals of 3 to 6 months are recommended as a key component of proactive oral healthcare.
